# The *Salmonella* phage shock protein system is required for defense against host antimicrobial peptides

**DOI:** 10.1371/journal.ppat.1013132

**Published:** 2025-09-09

**Authors:** Marie-Ange Massicotte, Aline A. Fiebig, Andrei Bogza, Brian K. Coombes

**Affiliations:** 1 Department of Biochemistry and Biomedical Sciences, McMaster University, Hamilton, Ontario, Canada; 2 Michael G. DeGroote Institute for Infectious Disease Research, McMaster University, Hamilton, Ontario, Canada; 3 Department of Microbiology and Immunology, McGill University, Montreal, Québec, Canada; 4 McGill Centre for Microbiome Research, McGill University, Montreal, Québec, Canada; University of the Chinese Academy of Sciences, CHINA

## Abstract

Macrophages are professional phagocytes that play a major role in engulfing and eliminating invading pathogens. Some intracellular pathogens, such as *Salmonella enterica* serovar Typhimurium, exploit macrophages as niches for their replication, which requires precise and dynamic modulation of bacterial gene expression in order to resist the hostile intracellular environment. Here, we present a comprehensive analysis of the global transcriptome of *S.* Typhimurium across four stages of infection of primary macrophages. Our results revealed a profound change in early-stage gene expression dominated by pathways linked to metabolic processes required for *Salmonella* adaptation to the proinflammatory conditions of the macrophage. We identified the phage shock protein (Psp) system to be highly expressed in intracellular *S.* Typhimurium, with sustained high expression over the course of infection. We determined that the Psp system is regulated by the virulence-associated two-component system SsrA-SsrB, which coordinates its expression with critical bacterial functions required for immune evasion and intracellular survival. Functional assays demonstrated that the Psp system mediates resistance to host antimicrobial peptides, including cathelicidin-related antimicrobial peptide (CRAMP), which we demonstrate supports bacterial persistence in host tissues and survival within macrophages. Our findings establish the Psp system as a new and critical adaptive mechanism for evading host immune defenses and highlight the utility of temporal transcriptomics in unraveling the genetic strategies employed by *S.* Typhimurium during macrophage infection.

## Author summary

*Salmonella enterica* is an important global pathogen that infects a wide range of mammalian hosts, requiring it to survive in diverse and hostile environments. A key aspect of *Salmonella* pathogenesis is its ability to reside within host immune cells like macrophages, where it must rapidly adapt to intracellular conditions. To do so, the bacteria must ensure correct spatiotemporal expression of virulence genes to maximise fitness in each environment. To better understand how *Salmonella* modulates its gene expression during infection of host cells, we defined its transcriptome at four distinct stages of primary murine macrophage infection. Our findings reveal that the first stage of early infection is dominated by changes in gene expression of metabolic circuits. Furthermore, we identified the phage-shock protein (Psp) system as highly expressed within intracellular *Salmonella* throughout the course of infection as a result of regulatory evolution that coordinates its expression with virulence genes. We showed that this system is required for bacterial survival within macrophages and host tissues by mediating resistance to host cationic antimicrobial peptides. These findings highlight the dynamic nature of *Salmonella’s* transcriptional response during macrophage infection and uncover a previously unknown function of the Psp system as an adaptive mechanism for evading host immune defenses.

## Introduction

*Salmonella enterica* serovar Typhimurium (*S.* Typhimurium) is a leading cause of gastrointestinal disease worldwide [1]. Although enteric salmonellosis is generally self-limiting, infections can escalate to life-threatening systemic illnesses in immunocompromised individuals or when caused by emerging invasive non-typhoidal *Salmonella* (iNTS) strains [2,3]. The efficacy of antibiotic therapy has been consistently compromised by the emergence of multidrug resistant strains [4,5]. Addressing this global health challenge requires a deeper understanding of the virulence mechanisms underlying *S.* Typhimurium pathogenesis to identify novel targets for anti-*Salmonella* therapies.

A hallmark of *S.* Typhimurium pathogenesis is its capacity to evade the innate immune response and establish a replication niche within innate immune cells, particularly macrophages. Intracellular replication occurs within a specialized phagosome called the *Salmonella*-containing vacuole (SCV) [6] where *Salmonella* resists phagosomal destruction while accessing nutrients to support replication and survival [7,8]. Infected macrophages can serve as reservoirs for bacterial dissemination to systemic sites [9,10]. Despite offering a replicative niche, the SCV presents several antimicrobial challenges, including an acidified microenvironment, restriction of essential ions and metals, and exposure to toxic compounds such as cationic antimicrobial peptides (cAMPs), reactive oxygen species (ROS), and reactive nitrogen species (RNS) [11]. To overcome these barriers, *S.* Typhimurium employs tightly regulated gene networks that integrate signals originating from the innate immune response with transcriptomic changes necessary for intracellular survival [12–14].

Despite decades of study, the mechanisms underlying *S.* Typhimurium’s evasion of host defenses and the gene networks that underpin these processes remain incompletely understood. Early microarray studies provided foundational insights into *S.* Typhimurium gene expression during macrophage or epithelial cell infections [15,16]. However, these approaches lacked the resolution that deep-sequencing technologies now offer [17]. RNA sequencing (RNA-seq) has been used to define the primary transcriptome of *S.* Typhimurium during macrophage infection, but these studies have relied on single time-point analyses [18], precluding an understanding of the temporal transcriptional response. Recently, innovative work employing a promoter-reporter library has shed new light on the temporal transcriptional dynamics of intramacrophage *S.* Typhimurium throughout the course of infection [19]. Nevertheless, most intracellular transcriptomics studies have used immortalized cell lines such as RAW264.7, J774A.1, or HeLa cells, which only partially recapitulate the biological processes occurring in primary cells [20]. Investigations using primary cell models are required to uncover the transcriptional strategies employed by *S.* Typhimurium in biologically relevant contexts [21].

Here, we examined the transcriptional response of *S.* Typhimurium at four distinct stages of infection in primary bone marrow-derived macrophages. We found that the most extensive transcriptional changes occurred during the early stages of infection, particularly within the first four hours. Among the genes most highly upregulated during this period, the phage shock protein (Psp) system, an envelope stress response pathway critical for mitigating inner membrane damage, emerged as a key transcriptional response. We demonstrated that the major phenotypic output of Psp system activation is bacterial resistance to host antimicrobial peptides and survival within macrophages, and that this regulation appears to have been selected via cis-regulatory evolution for co-expression with other major virulence factors. Together, these results provide new insights into the temporal transcriptional adaptations of *S.* Typhimurium during macrophage infection and establish a novel role for the Psp system in bacterial resistance to host antimicrobial defenses.

## Results

### Intracellular *S.* Typhimurium undergoes rapid transcriptional changes during the early stages of macrophage infection

**Fig 1:**
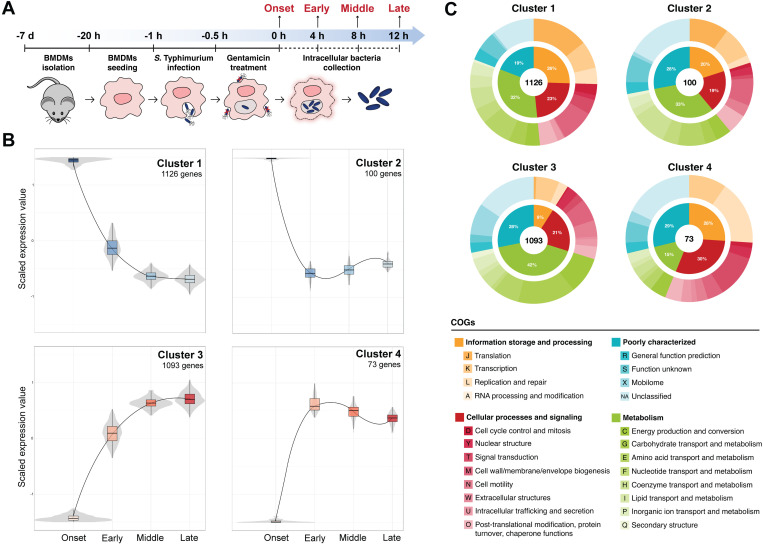
*S.* Typhimurium transcriptional profile changes drastically between onset and early stages of macrophage infection. (A) Schematic representation of the experiment. (B) Clusters of significantly differentially regulated genes in S. Typhimurium during the infection of macrophages identifies four patterns of expression over time. Genes are plotted on the y-axis according to a scaled expression value (z-score). For each cluster, volcano and box plots were constructed for each time point, with median expression level denoted by the horizontal line. Lines connect the mean expression levels of consecutive time points, to display the gene expression trend of that cluster. Genes that did not match the expression profiles of any of the clusters were omitted. Clustering was performed using DEGreport with genes of an adjusted P value < 0.01 calculated by the LTR test of DESeq2. Data are from three biologically independent replicates (N = 3). (C) All differentially expressed genes identified in (B) were assigned to a COG functional category. Percentages reflect the abundance of each major COG category (inner circle) in each cluster. As in (B); N = 3. The mouse clipart image in Fig 1 is open source from OpenClipart.org (https://openclipart.org/detail/17558/simple-cartoon-mouse). All other icons and graphics were hand-drawn by us and are original.

The macrophage niche is a highly dynamic environment to which *S.* Typhimurium must continuously adapt for intracellular survival. To assess the extent of transcriptional changes in intracellular *S.* Typhimurium, we analyzed its transcriptome at four stages of infection in primary macrophages that we defined as onset, early, middle, and late (Fig 1A). The stages were based on previous literature [15] and reflect key events encountered by *S.* Typhimurium within the SCV, including phagosome acidification [22], oxidative and nitrosative stress [23,24], SCV maturation [25], and exposure to cAMPs [26]. Bone marrow-derived macrophages (BMDMs) from C57BL/6J mice were infected with wild-type *S.* Typhimurium, and RNA from intracellular bacteria was extracted at 0, 4, 8, and 12 h post-infection [15,18]. cDNA libraries reached a sequencing depth of ~ 20 million reads that mapped to the *Salmonella* reference genome for ~ 600x genome coverage for each library [27,28].

To identify *S.* Typhimurium genes with significant changes in expression across the different stages of infection, we performed differential expression analysis using the Likelihood ratio test (LRT) in DESeq2. This method, ideal for comparative analysis within a time series [29], identified 2,386 differentially expressed genes throughout macrophage infection, representing nearly half of the *S.* Typhimurium genome. Clustering analysis of significant genes revealed four expression patterns, the majority of which fell into Cluster 1, representing peak expression at onset (1,126 genes) or Cluster 3 representing peak expression at late infection (1,093 genes) (Fig 1B, S1 Table). These data highlight a striking transcriptional shift during the transition from onset to early infection, suggesting a crucial period of widespread genetic reprogramming as an adaptive response to the intracellular environment. Building on previous microarray studies [13], these data indicate that early infection represents a pivotal stage of S. Typhimurium adaptation and survival within host macrophages.

To investigate the biological processes reflected within each expression cluster, genes were assigned to clusters of orthologous groups (COGs) categories (Fig 1C, S1 Table). Genes with decreasing expression over time (clusters 1 and 2) were predominantly linked to energy-intensive processes and rapid growth. For example, we observed decreased expression of genes involved in ribosome biogenesis, including the *rps* and *rpl* operons [30], the *rsgA* and *rim* operons encoding accessory proteins involved in ribosome assembly and maturation, and the *rsm* genes responsible for rRNA modifications. In line with this, genes involved in cell division, *fts* and *zip*, shared this expression pattern [31]. Interestingly, several ROS detoxifying enzymes, including *katG*, *sodA*, *sodCII*, and *ahpC* [32], grouped into expression clusters 1 and 2, suggesting that *S.* Typhimurium resists an early respiratory burst that subsides as the infection progresses. Together, these data indicated that *S.* Typhimurium transitions from early, active replication to energy conservation that supports survival in the host macrophage.

In contrast with this metabolic reprogramming, our data revealed that genes whose expression increases over time (clusters 3 and 4) are predominantly associated with alternative respiratory metabolism and adaptation to nutrient scarcity. Genes with dominant expression in these categories included enzymes required to sustain anaerobic respiration, such as the nitrate reductases *nar* and *nap* operons [33], the *ttr* operon encoding a tetrathionate reductase [34], the *hyp* operon encoding for an electron donor NiFe hydrogenase for fumarate respiration mediated by a fumarate reductase encoded by *frdABCD* [35], and the *dmsABC* operon which encodes for the three subunits of the anaerobic dimethyl sulfoxide reductase [36]. Additionally, we detected the upregulation of several genes involved in lipid and sugar metabolism previously shown to be important for replication in mice and macrophages [37,38]. This included the *eut* operon, required for the utilization of ethanolamine as a source of carbon and nitrogen and as an environmental cue used by *S.* Typhimurium to coordinate metabolism and virulence [39,40]. We also detected an upregulation of the *pdu* operon required for the degradation of 1,2-propanediol, the end-product of the anaerobic fermentation of fucose and rhamnose [41]. The genes involved in the degradation of fucose (*fuc* operon) and rhamnose (*rha* operon) were also upregulated in the late infection clusters [42]. Genes encoding components of the phosphotransferase system involved in the import and phosphorylation of various sugars like glucose, galactitol, mannitol, and fructose were upregulated [37]. These data suggest that in response to the oxygen and nutrient-depleted SCV, *S.* Typhimurium shifts toward anaerobic respiration via different terminal electron acceptors to support growth using host-derived nutrients as energy sources. Taken together, our temporal transcriptomic analysis highlights the importance of metabolic plasticity for *S.* Typhimurium to adapt and survive within the intracellular environment during macrophage infection.

### Time-course RNA sequencing reveals upregulation of the *psp* operon by intracellular *S.* Typhimurium

**Fig 2:**
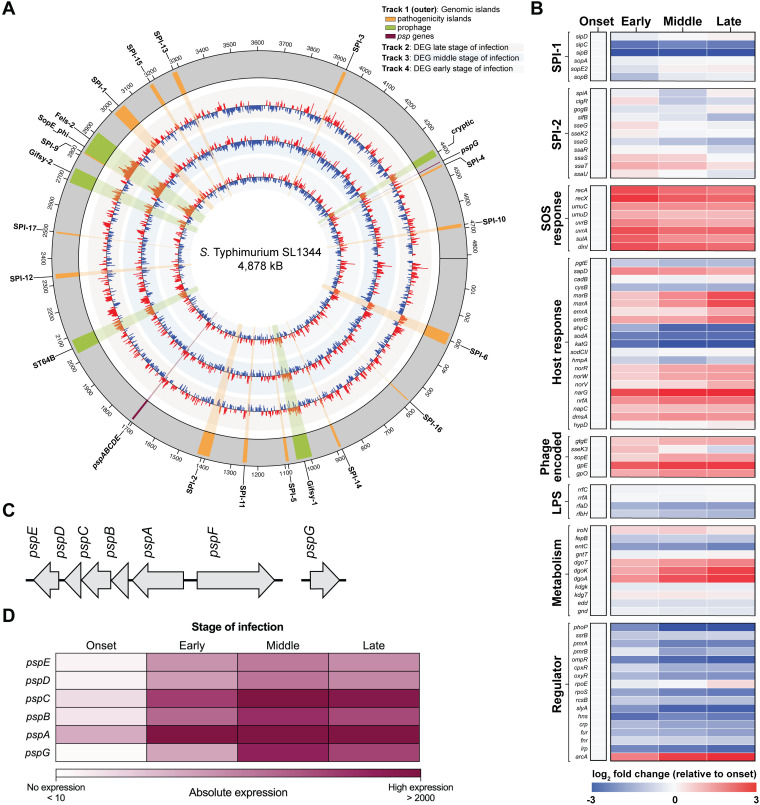
The *S.* Typhimurium *psp* operon is upregulated during the infection of macrophages. (A) Circos plot representing the genome-wide fold change in *S.* Typhimurium gene expression at early, middle, and late stages of infection relative to the onset stage from the inside to the outside tracks respectively. Red peaks indicate significantly up-regulated genes (log_2_ fold change >2), blue peaks indicate significantly down-regulated genes (log_2_ fold change > -2). Genes deemed not significantly regulated are not depicted. Genomic islands are indicated and labelled on track 1 (outermost circle), where pathogenicity islands are in orange, prophage in green, and *psp* genes are in purple. Data are from three biologically independent replicates (N = 3). (B) The expression of individual genes shown as fold-change for early, middle, and late stages of infection relative to onset of infection. Relevant SPI-1 and SPI-2 genes were selected along with genes known to regulate the SOS response, detoxification of the SCV, LPS synthesis, and various metabolic systems. Relative expression of phage-encoded genes and key transcriptional regulators for *S.* Typhimurium pathogenesis are also shown. All data is the mean of three biological replicates (N = 3). (C) Schematic representation of the *psp* operon in S. Typhimurium. (D) Heatmap showing expression of S. Typhimurium *psp* genes that are upregulated during the onset, early, middle, and late stages of macrophage infection. The heatmap colors represent the absolute expression levels (TPM values) based on the color bar below. Data are from three biologically independent replicates (N = 3).

Our clustering analysis revealed that *S.* Typhimurium undergoes substantial transcriptional changes during the onset and early stages of macrophage infection. To quantify these changes, we performed differential expression analysis of *S.* Typhimurium genes at early, middle, and late stages of infection relative to the onset stage using DESeq2 (Fig 2A and S2 Table) [29]. Approximately 50% of *S.* Typhimurium genes were differentially regulated in at least one stage of infection compared to onset, consistent with the results of the clustering analysis. As expected, most genes exhibited only moderate changes in expression across early, middle, and late stages of infection relative to onset, underscoring a model in which initial transcriptional shifts are pivotal for *S.* Typhimurium intracellular survival within the hostile SCV environment.

A hallmark of *S.* Typhimurium pathogenesis is the coordinated regulation of virulence genes encoded on *Salmonella* pathogenicity islands (SPIs). The *S.* Typhimurium SL1344 genome contains over 15 SPIs [43,44], with SPI-1 and SPI-2 being central to invasion and intracellular survival, respectively [45]. In accordance with their distinct roles, SPI-1 and SPI-2 are differentially regulated and precise transcriptional signatures have been documented [18,46]. In line with established models, we observed the downregulation or moderate change in expression of key SPI-1 genes following bacterial internalization into macrophages, such as *sipB*, *sipC*, and *sopB.* Surprisingly, we did not detect strong changes in the expression of SPI-2 genes across the time course of infection (Fig 2B). This likely reflects their already high expression at the onset of infection, and the observed moderate variation over time supports the idea that SPI-2 genes are continuously required for intracellular *S.* Typhimurium to maintain the SCV and promote survival.

In parallel with virulence gene regulation, *S.* Typhimurium must mitigate the damaging effects of various host-derived immune factors within the SCV. A striking transcriptional feature of our data was the robust induction of the SOS response, exemplified by the upregulation of *recA*, *umuCD*, and *uvrAB*, which peaked at the early stage of infection (Fig 2B). This suggests exposure to DNA damaging agents, most likely resulting from the oxidative burst that occurs immediately upon macrophage uptake which is a potent inducer of bacterial SOS responses [15,47]. Supporting this, the OxyR regulator and genes involved in oxidative stress defenses (*ahpC*, *sodA*, *katG*, *sodCII*) were strongly downregulated as infection progressed [32,48], suggesting that ROS exposure peaks early and diminishes over time, consistent with the results of the clustering analysis. In contrast, the induction of nitric oxide detoxification genes (*hmpA*, *norR*, *norV*, *norW*) implies a shift toward nitrosative stress as the dominant free radical following onset of infection (Fig 2B) [49].

Our analysis also uncovered broad upregulation of genes encoded on prophages during infection, including Gifsy-1, Gifsy-2, ST64B, and Fels2/SopEΦ (Fig 2A) [50]. This observation aligns with prior reports showing that prophage loci are transcriptionally active within macrophages [19], and likely reflects the high levels of DNA damage from exposure to genotoxic compounds, triggering prophage induction [50]. Prophage induction can positively contribute to *S.* Typhimurium virulence through the expression of phage-encoded effectors. Notably, several of these, including *gtgE* (from Gifsy-2) [51], *sseK3* (from ST64B) [50], *sopE* (from Fels2/SopEΦ) [52], have established roles in promoting survival within the host and overall pathogenesis [53].

Adaptation to the evolving SCV environment also involved dynamic metabolic reprogramming (Fig 1C). A sharp induction of the genes mediating gluconate and galactonate catabolism (*dgoT*, *dgoK*, and *dgoA*) highlights the exploitation of alternative carbon sources during intracellular growth (Fig 2B) [54]. Meanwhile, downregulation of the Fur-regulated iron acquisition genes *entABCDE* and *fepB*, suggests that *S.* Typhimurium is not deprived of iron (Fe^2+^) within the SCV [55]. The induction of multiple anaerobic respiratory operons, including *nar*, *nrf*, *nap*, *dms*, and *hyp* [33,36], alongside the upregulation of the global regulator ArcA [56], suggests a transition to anaerobic metabolism driven by declining oxygen availability in the SCV (Fig 2B). This is consistent with our clustering analysis data, which highlighted the switch to alternative elector acceptors as infection progresses.

Interestingly, while many of these stress response and metabolic systems remain active beyond the onset phase of infection, expression of key global regulators, including PhoP, PmrA, and OmpR, was markedly reduced in later stages. This suggests that their roles are most critical during the initial encounter with macrophages, where they orchestrate early transcriptional responses necessary for intracellular adaptation. Their diminished expression was previously noted [15], and may reflect a shift toward regulator-independent maintenance of the intracellular program once key survival mechanisms are in place. Together, these data emphasize that the intracellular lifestyle of *S.* Typhimurium is underpinned by a tightly regulated, multiphasic transcriptional response that enables the pathogen to establish a replicative niche, mitigate host defenses, and persist within hostile cellular environments.

Finally, as a means to identify new infection biology, we screened operons for genes that were significantly upregulated across all stages of infection following infection onset. As a result, we identified the entire phage shock protein (*psp*) system as highly expressed throughout infection, suggesting a pivotal role in supporting intracellular infection (Figs 2D, S1). The Psp system is an envelope stress mechanism encoded by the *psp* operon (*pspABCDE*), the unlinked *pspG* gene, and the transcriptional activator *pspF* located upstream of *pspA* (Fig 2C). Studies in *Escherichia coli* have shown that the Psp system is activated by damage to the inner membrane or loss of the proton motive force (PMF). Upon induction, PspA preserves the PMF, while the inner membrane PspBC complex mitigates toxicity from mislocalized secretins [57,58]. Currently, little is known about the biological functions of the other Psp proteins. Although the expression of *psp* genes in *S.* Typhimurium has been reported during infection of epithelial cells and macrophages [15,16,18], the physiological significance of this upregulation for its pathogenesis has not been elucidated.

### The transcriptional regulator SsrB controls the expression of the Psp system

**Fig 3:**
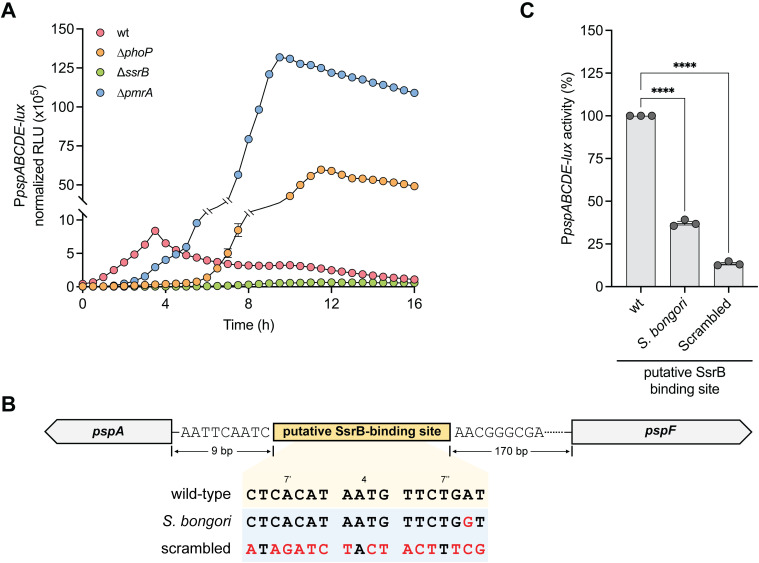
The Psp system is transcriptionally regulated by SsrB. (A) Transcriptional reporter of the *pspABCDE* operon promoter in ∆*phoP*, ∆*pmrA*, ∆*ssrB*, or wild-type (wt) *S.* Typhimurium strains grown in infection-mimicking media (LPM). Data are mean relative light units (RLU) normalized to the optical density of the culture from three independent experiments (N = 3). (B) Graphical representation of the putative SsrB-binding site identified upstream of the *pspA* gene. The sequences of the wild-type SsrB-binding motif and mutated binding sites from the *psp* promoter are shown. The bases colored in red are used to highlight the mutated bases in putative *S. bongori* and scrambled SsrB-binding site relative to wild-type. (C) Transcriptional reporter data for the wild-type (wt) and the two mutated SsrB-binding site as defined in (B). Promoter activity was measured as the ratio of RLU/optical density normalized to the promoter activity from the wild-type reporter at 4 h. Data are the means standard error of the mean from three independent experiments (N = 3). Groups were compared against wild-type via one-way ANOVA. ****p < 0.0001 (Holm-Sidak’s multiple comparisons test).

*S.* Typhimurium relies on a complex regulatory network of two-component regulatory systems (TCSs) to coordinate the expression of virulence genes essential for intracellular survival [59]. Given the strong upregulation of *psp* genes during macrophage infection, we considered the possibility that the Psp system in *Salmonella* was regulated by a TCS active in the intracellular niche. Specifically, we investigated PhoQ-PhoP, PmrB-PmrA, and SsrA-SsrB, which are known to regulate *S.* Typhimurium’s intracellular fitness and virulence features [60–62]. To assess the involvement of these TCS, we constructed a luciferase transcriptional reporter of the *pspABCDE* promoter (P*pspABCDE*-lux) and monitored promoter activity over 16 h in wild-type *S.* Typhimurium and strains with deletions of *phoP*, *pmrA*, or *ssrB* (∆*phoP*, ∆*pmrA*, ∆*ssrB*). These assays were performed in an acidic, low-phosphate, low-magnesium medium (LPM) that was established to mimic conditions of the SCV [18,46,63]. Deletion of *phoP* or *pmrA* significantly increased *psp* promoter activity compared to wild type (Fig 3A), consistent with earlier findings in a *rpoE* mutant [64]. In contrast, the deletion of *ssrB* completely abolished *psp* promoter activity, establishing the virulence-associated SsrA-SsrB TCS as a critical regulator of the Psp system.

SsrB has been shown to capture a large genetic network in *S.* Typhimurium that integrates both ancestral and horizontally acquired genes via regulatory evolution [44,65]. To determine if the Psp system in *Salmonella* has undergone regulatory evolution and selection for SsrB control, we scanned the intergenic region upstream of *pspA* for evidence of the flexible 18 bp palindrome sequence that defines DNA recognition by SsrB (S2 Fig). This analysis identified a putative SsrB binding site 9 bp upstream of the transcriptional start site of *pspA* (Fig 3B). To validate this site as an input sequence for SsrB regulation, we designed two transcriptional reporters; one replacing the SsrB binding site with the homologous sequence from *S. bongori,* which lacks the SsrA-SsrB TCS but contains an otherwise identical *psp* operon, and a second reporter with a scrambled version of the putative SsrB binding sequence in which the 18 bp palindrome was randomized (Fig 3B). The strain containing the *S. bongori* replacement sequence, with only a single nucleotide replacement, reduced *psp* promoter activity to ~35% of wild-type levels, whereas the scrambled binding site produced only ~12% residual activity relative to wild-type (Figs 3C and S2). Taken together, these data established that the Psp system is regulated by the SsrA-SsrB TCS in *S.* Typhimurium and implied an important role for this system in intracellular survival in macrophages.

### The Psp system promotes *S.* Typhimurium survival in primary macrophages and contributes to its persistence in host tissues

**Fig 4:**
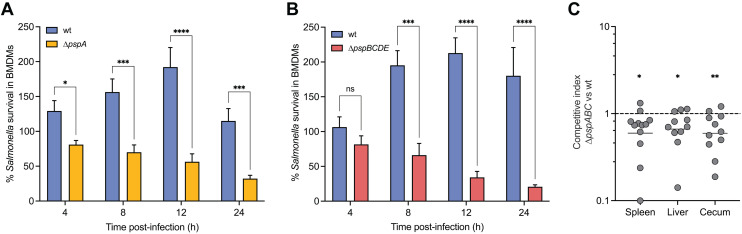
Psp-deficient *S.* Typhimurium bacteria are attenuated for virulence in primary macrophages and *in vivo.* (A) *S.* Typhimurium ∆*pspA* bacteria show increased susceptibility to killing by primary macrophages compared to wild-type (wt) bacteria. Survival was measured as the intracellular bacterial burdens enumerated at 4, 8, 12, and 24 h following infection normalized to the values of the initial number of internalized bacteria (T0). Bar plots depict mean of at least four biological replicates (N ≥ 4) and error bars indicate standard error of the mean. Groups were compared by two-way ANOVA, *p < 0.05, ***p < 0.001, ****p < 0.0001 (Holm-Sidak’s multiple comparisons test). (B) As in (A), but with the Δ*pspBCDE* mutant strain. *S.* Typhimurium Δ*pspBCDE* bacteria show an intramacrophage survival defect relative to wild-type (wt) bacteria. Bar plots depict mean of four biological replicates (N = 4) and error bars indicate standard error of the mean. Groups were compared by two-way ANOVA, ***p < 0.001, ****p < 0.0001 (Holm-Sidak’s multiple comparisons test). (C) Wild-type *S.* Typhimurium out-competed the ∆*pspA* mutant in all gut tissues. C57BL/6J mice were infected by intraperitoneal injection with equal number of wild-type *S.* Typhimurium and ∆*pspABC* mutant strains, and the competitive index was calculated after 2 days of infection. Each data point represents the value for an individual animal, and the horizontal lines indicate geometric means. The broken line shows a competitive index of 1, representing equal fitness. Groups were compared against a value of 1 using one-sample parametric T-test, *p < 0.05, **p < 0.01. Data are from two independent experiments (N = 2).

The upregulation of *psp* genes during macrophage infection, and the apparent regulatory evolution of this system for SsrB control, strongly suggested that the Psp system plays a critical role in intracellular *S.* Typhimurium pathogenesis. To isolate the impact of the Psp response on bacterial intracellular fitness, we generated two isogenic mutants, one with a deletion of *pspA* (∆*pspA*) and the other with a deletion of *pspBCDE* (∆*pspBCDE*) and validated that both mutants were not inherently growth defective in both rich and infection-mimicking media (S3 Fig). To assess whether Psp deficiency affected bacterial survival in macrophages, we infected BMDMs isolated from C567BL/6J mice with either wild-type S. Typhimurium, ∆*pspA*, or ∆*pspBCDE* and monitored bacterial survival over time. In the first 12 h after infection, there was a 200% increase in intracellular wild-type bacteria, whereas survival of the ∆*pspA* mutant was only ~50% and significantly decreased further to 30% after 24 h of infection (Fig 4A). Mirroring this phenotype, only ~20% of the ∆*pspBCDE* mutant had survived after 24 h of infection, while the number of intracellular wild-type bacteria nearly doubled (Fig 4B). These findings indicate that PspA and PspBCDE are required for *S.* Typhimurium survival and replication in primary macrophages, consistent with the elevated expression of the Psp system during intracellular infection.

To evaluate whether the Psp system contributes to *S.* Typhimurium fitness during *in vivo* infection, we constructed a ∆*pspABC* mutant strain in SL1344 and assessed its infection potential in C57BL/6J mice. To do this, we performed competitive infections between the ∆*pspABC* mutant and wild-type bacteria and quantified bacterial burden two days after infection in the spleen, liver, and cecum. In all three tissues, wild-type bacteria significantly out-competed the ∆*pspABC* mutant (CI, spleen; 0.7043, P < 0.05, CI liver; 0.7453, P < 0.05, and CI cecum; 0.6779, P < 0.01) (Fig 4C). These results are consistent with those of a previous report [66], and demonstrate that Psp-deficient *S.* Typhimurium is attenuated for virulence *in vivo*. The importance of the Psp system for *in vivo* survival, and intracellular fitness, is not restricted to C57BL/6J mice, a more susceptible mouse model of *S.* Typhimurium infection due to the lack of functional *NRAMP1* gene (NRAMP^-^) [67]. We found that Psp-deficient *S.* Typhimurium was also defective for survival in NRAMP1^+^ macrophages (S4A Fig) and in competitive infection against wild-type bacteria in NRAMP1^+^ mice (S4B Fig). Together, these results established that the Psp system contributes to bacterial fitness in both primary macrophages and mouse models of infection.

### The Psp system mediates resistance to the murine antimicrobial peptide CRAMP

**Fig 5:**
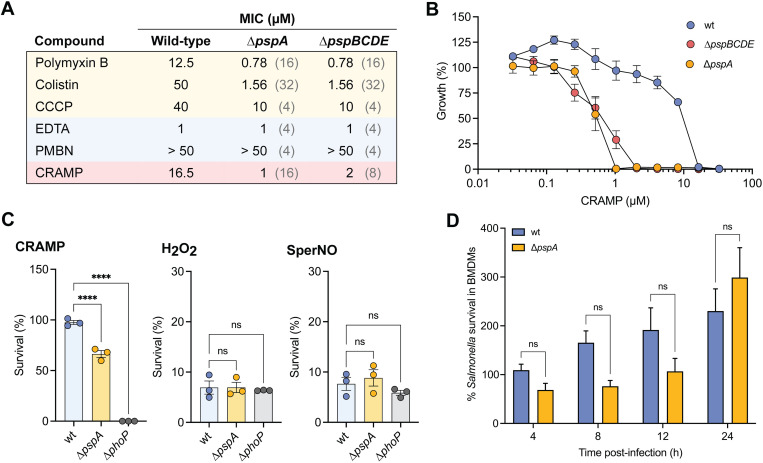
PspA is required for resistance to CRAMP. (A) Table of MIC values for various PMF disruptors (polymyxin B, colistin, and CCCP) and outer-membrane disruptors (EDTA, and polymyxin B nonapeptide; PMBN) against wild-type *S.* Typhimurium, ∆*pspA* mutant, and ∆*pspBCDE* mutant in infection-mimicking media (LPM). Fold-change values are written in light grey. The ∆*pspA* and ∆*pspBCDE* mutants shows increased susceptibility to all the PMF disruptors tested relative to wild-type, but not the outer-membrane disruptors. Data are representative of three biological replicates (N = 3). (B) Growth of wild-type *S.* Typhimurium, ∆*pspA* mutant, and ∆*pspBCDE* mutant in the presence of CRAMP in infection-mimicking media (LPM). Data is from three biological replicates (N = 3), dots and error indicate mean and standard error of the mean. (C) *S.* Typhimurium ∆*pspA* mutant is more susceptible to killing by CRAMP than the wild-type (wt) is, but not to hydrogen peroxide (H_2_O_2_) nor Spermine NONOate (SperNO) in infection-mimicking media (LPM). Bacteria were incubated with 10µM of CRAMP or 50µM of H_2_O_2_, or 50µM of SperNO for 3 h. *S.* Typhimurium ∆*phoP* mutant was used as a control for extreme sensitivity to antimicrobial peptides. Bar plots depict mean of three biological replicates (N = 3) and error bars indicate standard error of the mean. Groups were compared against wild-type via one-way ANOVA. ****p < 0.0001 (Holm-Sidak’s multiple comparisons test); ns, not significantly different. (D) Intracellular replication defect of *S.* Typhimurium ∆*pspA* bacteria is abrogated in *Camp*^-/-^ macrophages. As in Fig 3A, survival was measured as the ratio of the number of viable bacteria enumerated at 4, 8, 12, and 24 h compared to the initial number of internalized bacteria at T0. Bar plots depict mean of three biological replicates (N = 3) and error bars indicate standard error of the mean.

To investigate how the Psp system supports *S.* Typhimurium survival during infection, we mainly focused on the functional role of PspA. In *E. coli*, the Psp system, particularly PspA, is thought to preserve the PMF during extracytoplasmic stress [68,69]. In *S.* Typhimurium, while the physiological role of Psp proteins remains poorly understood, PspA has been implicated in divalent metal transport [70], resistance to the proton ionophore CCCP, and survival under stress conditions such as stationary phase and resistance to the innate immune protein bactericidal permeability-increasing protein (BPI) in *S.* Typhimurium lacking the alternative sigma factor RpoE [64]. Given the association of these conditions with PMF disruption, we first tested the susceptibility of a ∆*pspA* mutant to PMF-disrupting agents [71,72]. For this, we determined the minimum inhibitory concentrations (MIC) of polymyxin B, colistin (polymyxin E), and CCCP for the wild-type and ∆*pspA* mutant strains in infection-mimicking media (LPM). The ∆*pspA* mutant showed significantly increased susceptibility compared to the wild-type, with a 16-fold, 32-fold, and 4-fold increase in susceptibility to polymyxin B, colistin, and CCCP, respectively (Fig 5A). To confirm that the mutant’s susceptibility was specific to PMF disruption and not general membrane damage, we tested MICs of EDTA and polymyxin B nonapeptide (PMBN), which both disrupt the outer membrane without causing direct damage to the inner membrane [73,74]. No differences in MICs were observed between the wild-type and ∆*pspA* mutant for these agents (Fig 5A), confirming that PspA confers resistance to PMF-disrupting agents likely through inner membrane stabilization. To begin exploring whether *S.* Typhimurium PspB, PspC, PspD, and PspE also contribute to resistance against PMF destabilization, we determined the MICs of polymyxin B, colistin, CCCP, EDTA, and PMBN for a ∆*pspBCDE* mutant. The ∆*pspBCDE* strain was significantly more susceptible to all three inner-membrane destabilizing agents tested, while its susceptibility to outer-membrane permeabilizing compounds remained unchanged relative to wild-type (Fig 5A). These findings parallel those observed for the ∆*pspA* mutant and suggest that the entire Psp system is required for resistance to inner membrane-disrupting stresses.

Next, we investigated the protective role of the Psp system against host-derived antimicrobial factors. Among these, cationic antimicrobial peptides (cAMPs) are important host defense mechanisms that can target the bacterial cytoplasmic membrane to induce cell lysis [75–78]. In murine macrophages, the primary cAMP is cathelicidin-related antimicrobial peptide (CRAMP), a homolog of human LL-37 [79]. Infections with different intracellular bacteria have been shown to induce CRAMP upregulation [80,81], and CRAMP colocalizes with the SCV during *S.* Typhimurium infection [26]. MIC assays revealed that the ∆*pspA* and ∆*pspBCDE* mutants were, respectively, 16-fold and 8-fold more susceptible to CRAMP than wild-type (Fig 5B). Complementation of the ∆*pspA* mutant with *pspA* expressed from its native promoter completely restored resistance to CRAMP to wild-type levels (S5 Fig). Survival assays further demonstrated ∆*pspA* mutant susceptibility to CRAMP. After exposure to 10 μM CRAMP for 3 hours, ~ 60% of the ∆*pspA* mutant bacteria survived compared to nearly 100% survival for wild-type (Fig 5C). In contrast to the MIC values, the modest killing observed following CRAMP treatment is consistent with its previously reported bacteriostatic activity against *S.* Typhimurium, wherein bacterial growth is inhibited without causing rapid cell death [26]. As a control, we included a ∆*phoP S.* Typhimurium mutant which has extreme sensitivity to cAMPs [82,83]. As expected, we observed the complete killing of the ∆*phoP* strain after 3 hours of CRAMP exposure. These results establish that PspA-deficient *S.* Typhimurium has a significant survival defect in the presence of CRAMP. To probe if PspA is important for resistance against other key host-derived antimicrobial factors found within the SCV, we repeated the survival assays in the presence of oxidative and nitrosative stressors. Survival assays using hydrogen peroxide and nitric oxide showed no significant differences between the ∆*pspA* mutant and wild-type (Fig 5C), suggesting that PspA’s protective role is more specific to AMP resistance.

Finally, to validate that PspA promotes *S.* Typhimurium survival within host macrophages by promoting resistance to CRAMP, we assessed bacterial survival in macrophages lacking CRAMP harvested from *Camp*^-/-^ mice. In contrast to infections in CRAMP-producing macrophages from C57BL/6J mice (Fig 4A), ∆*pspA* mutants showed no survival defect in *Camp*^-/-^ macrophages, with both mutant and wild-type strains exhibiting over 200% increase in viable bacteria after 24 hours (Figs 5D, S6). These findings confirm that the fitness defect of PspA-deficient *S.* Typhimurium in macrophages is specifically linked to increased susceptibility to CRAMP.

### PspA preserve bacterial membrane potential upon its disruption by CRAMP

**Fig 6:**
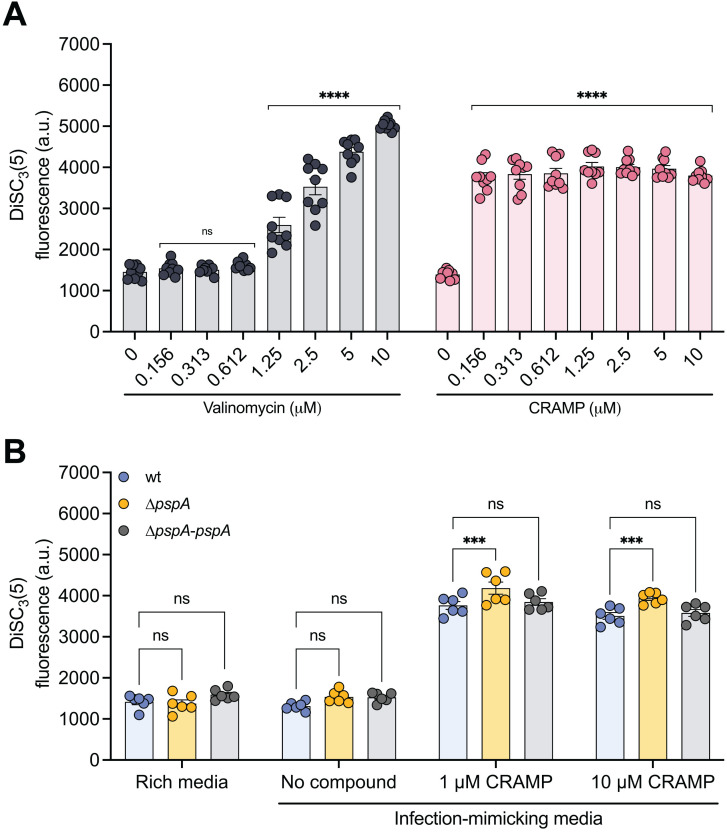
*S.* Typhimurium PspA helps maintain PMF upon its destabilization by CRAMP in infection-mimicking conditions. (A) CRAMP alters the PMF of *S.* Typhimurium. DiSC3(5) fluorescence in relative fluorescence units of wild-type bacteria in the presence of CRAMP or the membrane active control valinomycin. Bar plots depict mean of four biological replicates (N = 4) and error bars indicate standard error of the mean. Concentrations of each compound were compared against 0µM via two-way ANOVA. ****p < 0.0001 (Holm-Sidak’s multiple comparisons test); ns, not significantly different. (B) *S.* Typhimurium Δ*pspA* mutant as a lower PMF when exposed to CRAMP compared to wild-type (wt). Fluorescence intensity of DiSC3(5)-stained wild-type (wt), Δ*pspA* mutant, and Δ*pspA* mutant complemented with *pspA* (Δ*pspA-pspA*) strains exposed to 1µM or 10µM of CRAMP in riche media (LB) and infection-mimicking conditions (LPM). Bar plots depict mean of three biological replicates (N = 3) and error bars indicate standard error of the mean. Groups were compared against wt via two-way ANOVA. **p < 0.01 (Holm-Sidak’s multiple comparisons test); ns, not significantly different. Significance not shown on plot: ns, wt rich media vs wt no compound; ns, Δ*pspA* rich media vs Δ*pspA* no compound; ns, Δ*pspA-pspA* rich media vs Δ*pspA-pspA* no compound.

The PMF is critical to bacterial survival, driving vital cellular processes such as ATP synthesis, solute transport, and motility [84]. It is composed of two interdependent components, an electrical potential (∆ψ) and a proton gradient (∆pH), which together establish the electrochemical gradient across the inner membrane of Gram-negative bacteria. As dissipation of either ∆ψ or ∆pH results in a collapse of the PMF and loss of essential cellular functions, bacteria tightly regulate both components to maintain a stable PMF [71,85]. As previously discussed, a key physiological role of the Psp system is to preserve the PMF by preventing proton leakage across the inner membrane [69]. Based on this reported function and our findings that Psp-deficient mutants were compromised for survival against CRAMP, we hypothesized that CRAMP could disrupt bacterial PMF. Previous studies showed that CRAMP causes profound alterations to *S.* Typhimurium cytoplasmic structures without damaging the outer membrane [82], suggesting a potential PMF loss. To test whether CRAMP can disrupt bacterial PMF, we used 3,3’-dipropylthiadicarbo-cyanine iodide (DiSC_3_(5)), a fluorescent probe sensitive to changes in the PMF; disruption of the PMF leads to marked changes in fluorescence, whereas stable fluorescence indicates that the membrane potential is intact. Like our positive control, the ionophore valinomycin, CRAMP significantly altered DiSC_3_(5) fluorescence (Fig 6A), confirming that CRAMP can dissipate the PMF in *S.* Typhimurium.

Next, to determine whether PspA plays a role in mitigating PMF loss caused by CRAMP in *S.* Typhimurium, we monitored membrane potential in wild-type and ∆*pspA* mutant strains using the DiSC_3_(5) probe. In both rich and infection-mimicking (LPM) media, the two strains exhibited similarly low fluorescence, indicating intact membrane potential under baseline conditions and confirming that the absence of PspA alone does not compromise the PMF (Fig 6B). However, upon exposure to 1 µM or 10 µM CRAMP, we observed a sharp increase in fluorescence in both strains, consistent with our earlier findings that CRAMP dissipates the PMF. Notably, the ∆*pspA* mutant displayed significantly higher fluorescence than the wild-type following CRAMP treatment, suggesting a more pronounced collapse of the membrane potential. To further confirm that this phenotype was specifically due to the loss of *pspA*, we performed the same assays using the complemented strain (∆*pspA*-*pspA*), which restored the wild-type phenotype. These results support the model that PspA contributes to the maintenance of the PMF during inner membrane stress, and that in its absence, *S.* Typhimurium is less equipped to counteract CRAMP-induced PMF disruption.

## Discussion

The ability to precisely regulate gene expression in response to environmental cues is essential for *S.* Typhimurium’s intracellular survival, enabling adaptation to hostile immune challenges within the macrophage niche. Despite the importance of these transcriptional changes, which have mostly been defined at single time points during infection, less is known about the dynamic transcriptional response of *S.* Typhimurium over the course of infection in primary macrophages. By employing RNA sequencing to profile the bacterial transcriptome at four stages of primary macrophage infection, we revealed critical insights into the dynamics of *S.* Typhimurium gene regulation. Our data revealed that the most abundant transcriptional changes occur at the onset of infection, with gene expression stabilizing as the infection progresses. This transition reflects the bacteria’s rapid adaptation to the evolving macrophage environment, characterized initially by immune-driven stress and later by metabolic exploitation of host-derived nutrients.

### Metabolic adaptations underpinning intramacrophage survival

One of the key findings of our study is the identification of metabolic pathways supporting *S*. Typhimurium’s adaptation within macrophages. The upregulation of pathways for ethanolamine and propanediol metabolism underscores their importance as energy sources in the hypoxic macrophage niche. These findings expand on previous observations by demonstrating the critical role of host-derived nutrients in bacterial fitness under anaerobic conditions [40,41]. Moreover, our data highlight the activation of alternative electron acceptors, such as fumarate, nitrate, and dimethyl sulfoxide, which collectively support energy production in oxygen-limited environments [86–89]. Interestingly, the observed downregulation of reactive oxygen species (ROS)-detoxifying enzymes after the early infection stage suggests a metabolic shift towards glycolysis and alternative antioxidant defenses, emphasizing the metabolic flexibility of *S.* Typhimurium in countering oxidative stress [90,91]. In response to the important DNA damage caused by host-produced ROS, a strong SOS response is triggered by the bacteria, along with induction of diverse prophages which contributes to virulence and host adaptation [47,50]. Overall, this confirms and extends previous findings regarding *S.* Typhimurium’s ability to exploit metabolic resources offered by macrophages at different times during infection and adapt to the toxic intracellular environment [26,92].

### Novel role of the Psp system in intramacrophage survival

The survival of *S.* Typhimurium within macrophages depends on the expression of numerous genes, including those conferring resistance to host immune defenses. Among these, our transcriptomic analysis revealed strong induction of the *psp* genes. Upregulation of this system has been consistently observed in previous studies examining intracellular *S.* Typhimurium global gene expression across different *Salmonella* strains and host cell types [15,16,18,93,94]. The robust induction of *psp* genes appears to be unique to the intracellular environment, as the comprehensive transcriptomic dataset of *S.* Typhimurium grown under 16 *in vitro* conditions showed that none of the conditions tested triggered significant *psp* expression [46]. More recently, a study employing a promoter-reporter library of ~3,000 *S.* Typhimurium sequences monitored transcriptional activity during infection of RAW264.7 macrophages over 15 hours of infection [19]. Consistent with our RNA-seq data and other intracellular transcriptomic studies, *pspA* promoter was activated from 6 h post-infection, peaking at 9 h, and remaining active until 15 h. Although the activity signal of the *pspA* promoter was lower than that of the master transcriptional regulator *phoP*, it was comparable to *ssrAB*, the key regulator of SPI-2 genes expression. Thus, multiple lines of evidence, including the present study, support that *psp* gene upregulation is a conserved feature of *S.* Typhimurium adaptation to the intracellular niche.

Our study provides the first direct evidence that the Psp system mediates *S.* Typhimurium resistance to antimicrobial peptides, specifically CRAMP, within macrophages. Previous work suggested that PspA helps maintain energy homeostasis in bacterial cells, thereby indirectly supporting the function of divalent metal transporters required to compete against host Nramp1 [70]. Nevertheless, the upregulation of the Psp system in host cells lacking Nramp1 [15,16] suggested an alternative function. We demonstrated that, by preserving the PMF, the Psp system is essential for resisting CRAMP and other inner membrane destabilizing agents. This represents a significant advancement in understanding the Psp system’s role in bacterial pathogenesis, addressing a longstanding question regarding its contribution to *S.* Typhimurium fitness in *Nramp1*-lacking hosts. Our results also reveal that the loss of PspA renders *S.* Typhimurium significantly more susceptible to CRAMP-induced killing. This susceptibility was prevented in macrophages deficient in CRAMP production (*Camp*^-/-^), conclusively linking PspA to resistance against this host antimicrobial peptide. Moreover, our findings that PspBCDE collectively contribute to intracellular survival and resistance to PMF-disturbing agents, including CRAMP, provide the first insights into the physiological importance in *S.* Typhimurium. The specificity of the function reported here, combined with our evidence for the regulatory evolution of this system for intracellular expression by the SsrA-SsrB TCS, underscores the importance of the Psp system as a specialized AMP defense mechanism within the macrophage niche.

Future work is required to determine the precise mechanism by which PspA and other Psp proteins contribute to antimicrobial peptide resistance in intracellular *S.* Typhimurium. In *E. coli,* current evidence suggests that to mitigate inner membrane damage and maintain the PMF, PspA assembles into large oligomers that directly bind the inner surface of the cytoplasmic membrane. The resulting scaffold reduces inner membrane permeability which may also block proton leakage [69,95,96]. Given that PspA, along with PspF, are the most conserved Psp proteins within *Enterobacteriaceae* and constitute the Psp minimal system [97], it is reasonable to postulate that PspA might function in a similar fashion in *S.* Typhimurium. Stabilization of the inner membrane by PspA oligomers would help mitigate damage caused by CRAMP. Alternatively, it is also possible that the reinforced cytoplasmic membrane could prevent the entry of CRAMP into the cytoplasm. Exploring these hypotheses should be the focus of future research as it will bring further insight into the role and importance of the Psp system in *S.* Typhimurium pathogenesis.

### Regulation of the Psp System by SsrA-SsrB

A surprising discovery of our study is the regulatory connection between the Psp system and the SsrA-SsrB two-component system (TCS) in *S.* Typhimurium. While PhoP-PhoQ and PmrA-PmrB are traditionally regarded as the primary regulators of antimicrobial peptide resistance in *S.* Typhimurium [60,98], we found that these TCSs do not regulate the *psp* operon. Instead, SsrA-SsrB, a master regulator of intracellular survival [99,100], directly controls *psp* expression in *S.* Typhimurium through an evolved regulatory sequence in the intergenic region upstream of *pspA* that differs in the non-pathogenic *S. bongori*. This novel regulatory relationship highlights the adaptability of the SsrB regulon, which has evolved to capture a large number of ancestral and horizontally-acquired genes critical for bacterial fitness in the intracellular environment [65,101,102].

In summary, our study advances the understanding of the temporal transcriptional landscape of *S.* Typhimurium during macrophage infection, identifying novel metabolic adaptations and unveiling the critical role of the Psp system in resistance to antimicrobial peptides. Using temporal transcriptomics, we captured the first comprehensive global transcriptomic profile of intracellular *S.* Typhimurium since the pioneering microarray studies [15,16,103], overcoming the limitations of earlier low-throughput or single time-point analyses [18,93]. Our work complements recent targeted [104,105] and global promoter-reporter studies [19], as well as single-cell [106,107] and dual RNA-seq datasets [108,109], collectively deepening insight into how *S.* Typhimurium balances immune evasion and virulence with metabolic flexibility to thrive within the dynamic macrophage environment. Extending temporal transcriptomics to invasive strains or other host cell types, in combination with emerging approaches, will accelerate the discovery of new virulence mechanisms. Future research into the interplay between host defenses and bacterial stress responses will be instrumental in developing therapeutic strategies targeting either pathogen adaptations or macrophage immunity.

## Materials and methods

### Ethics statement

Animal experiments were conducted according to Canadian Council on Animal Care guidelines using protocols approved by the Animal Review Ethics Board at McMaster University under Animal Use Protocol #20-12-41.

### Bacterial strains and growth conditions

A detailed list of the strains and plasmids used in this study is provided in Table 1. All experiments were performed with *S.* Typhimurium *enterica* serovar Typhimurium strain SL1344. Routine propagation of bacteria was in LB media (10 g/L NaCl, 10 g/L Tryptone, 5 g/L yeast extract) supplemented with appropriate antibiotics (streptomycin, 100 μg/mL; chloramphenicol, 34 μg/mL; ampicillin, 200 μg/mL). Where indicated, bacteria were grown in LPM media (5 mM KCl, 7.5 mM (NH_4_)_2_SO_4_, 0.5 mM K_2_SO_4_, 80 mM MES pH 5.8, 0.1% (w/v) casamino acids, 0.3% (v/v) glycerol, 24 μM MgCl_2_, 337 μM PO_4_^3-^). Bacteria were grown at 37°C with shaking.

**Table 1:** Bacterial strains used in this study.

Strain	Source or reference
Wild-type *S.* Typhimurium strain SL1344	[110]
∆*pspA S.* Typhimurium strain SL1344	This study
∆*pspABC S.* Typhimurium strain SL1344	This study
∆*pspBDCE S.* Typhimurium strain SL1344	This study
∆*pspA S.* Typhimurium strain SL1344 + pGEN-MCS-*pspA*	This study
Wild-type *S.* Typhimurium strain SL1344 + pGEN-MCS	This study
∆*ssrB S.* Typhimurium strain SL1344	[44]
∆*phoP S.* Typhimurium strain SL1344	Coombes lab collection
∆*pmrA S.* Typhimurium strain SL1344	[111]
TOP10 *E. coli*	Invitrogen
Wild-type *S.* Typhimurium strain SL1344 + pGEN-P*pspABCDE-luxCDABE*	This study
∆*ssrB S.* Typhimurium strain SL1344 + pGEN-P*pspABCDE-luxCDABE*	This study
∆*phoP S.* Typhimurium strain SL1344 + pGEN-P*pspABCDE-luxCDABE*	This study
∆*pmrA S.* Typhimurium strain SL1344 + pGEN-P*pspABCDE-luxCDABE*	This study
Wild-type *S.* Typhimurium strain SL1344 + pGEN-P*pspABCDE-S.bongori-ssrB-luxCDABE*	This study
Wild-type *S.* Typhimurium strain SL1344 + pGEN-P*pspABCDE-scrambled-ssrB-luxCDABE*	This study
Wild-type *S.* Typhimurium strain SL1344 + pGEN-P*ssaB-luxCDABE*	[112]
Wild-type *S.* Typhimurium strain SL1344 + pGEN-empty-*luxCDABE*	[112]

### Mice

Six to eight-week-old female C57BL/6J (000664) and C3H/HeN (000659) mice were purchased from Jackson Laboratories and C57BL/6 cathelicidin-null (*Camp*^-/-^) mice were provided by E. Cobo (University of Calgary). Animals were housed in a specific pathogen-free barrier unit under Level 2 conditions. Mice were fed a regular chow diet *at libitum*.

### Cell culture maintenance

Cells were maintained in a humidified incubator at 37°C with 5% CO_2_. Bone marrow-derived macrophages (BMDMs) were differentiated from the marrow isolated from the femur and tibia of female C57BL/6, C3H/HeN, or *Camp*^*-/-*^ mice and maintained in RPMI containing 10% FBS (Gibco), 10% L929 fibroblast conditioned media, and 100 U penicillin-streptomycin. Cells were differentiated for 7 days in 150 mm Petri dishes, then lifted with ice-cold PBS for seeding in tissue culture-treated plates 20 h prior to infection. L929 fibroblast conditioned media was collected from the supernatants of L929 fibroblasts grown in DMEM with 10% FBS for 10 days.

### Cloning and mutant generation

Primers for cloning and mutant generation are listed in Table 2, and plasmids used in this study are listed in Table 3. All DNA manipulation procedures followed standard molecular biology protocols. Primers were synthesized by Sigma-Aldrich. PCRs were performed with Phusion, Phire II, or Taq DNA polymerases (ThermoFisher). All deletions and plasmid constructs were confirmed by PCR and verified by Sanger sequencing (McMaster Genomics Facility) or Whole Plasmid Sequencing (Plasmidsaurus, Oxford Nanopore Technology). In-frame, marked mutants of SL1344 *pspA*, *pspABC*, and *pspBCDE* were generated using Lambda Red Technology [113]. Wild-type SL1344 carrying pKD46 was transformed with linear PCR products containing gene-specific regions of homology and flanking the *cat* cassette carried by pKD3. Transformants were selected on LB agar supplemented with chloramphenicol (34 μg/mL) and knockouts were verified by PCR. To generate the *pspA* mutant complementation construct, the coding sequence of *pspA* and 800 bp upstream of the start codon was PCR-amplified and product was then cloned into pGEN-MCS after digesting with EcoRI/NotI (ThermoFisher). The sequenced-verified was transformed into ∆*pspA* S. Typhimurium strain SL1344 for expression. The transcriptional reporter constructs for P*pspABCDE* were generated by introducing the *pspABCDE* promoter into a luciferase reporter plasmid as previously described [114]. Briefly, ~ 500 bp regulatory region upstream of *pspABCDE* from SL1344 was PCR-amplified and cloned into the BamHI/SnaBI-digested pGEN-*luxCDABE* plasmid. The sequenced-verified plasmid was transformed into electrocompetent wild-type SL1344 or derivate strains.

**Table 2:** Plasmids used in this study.

Plasmid	Description	Reference
pKD3	Template plasmid for Lambda Red recombination	[113]
pKD46	Lambda Red recombinase expression plasmid	[113]
pGEN-*luxCDABE*	Lux transcriptional reporter plasmid	[115]
pGEN-MCS	Low-copy-no. cloning vector	[115]
pGEN-P*pspABCDE-luxCDABE*	Lux transcriptional reporter for *pspABCDE* promoter	This study
pGEN-P*pspABCDE-S.bongori-ssrB-luxCDABE*	Lux transcriptional reporter for *pspABCDE* promoter with putative SsrB-binding site from *S. bongori*	This study
pGEN-P*pspABCDE-scrambled-ssrB-luxCDABE*	Lux transcriptional reporter for *pspABCDE* promoter with scrambled putative SsrB-binding site	This study
pGEN-MCS-*pspA*	*pspA* with 800 bp upstream of coding sequence cloned into pGEN-MCS for complementation experiments	This study

**Table 3:** Primers used in this study.

Primer	Direction	Description	Sequence (5’ - 3’)
MAM3–28	F	∆*pspA* (flanking region 1)	tgcgttctccttacacctaaagctgatgagcgacggcgcgtatcggcgccgccattgtcattattgattatcttgcttcattttggctttgtgtaggctggagctgcttcg
MAM3–29	R	∆*pspA* (flanking region 2)	ttgtattaatcacatagcagggcatcgcccgttatcagaacattatgtgaggattgaattatgggtattttttctcgttttgccgacatcatatgaatatcctcctta
MAM3–32	F	∆*pspA* (upstream)	atggttaacgggatggccag
MAM3–33	R	∆*pspA* (downstream)	cgggagacttgttccagtac
MAM3–55	F	∆*pspABC* (flanking region 1)	ttcaccttctgcccggcgcgttgccagcgagtattcatcttcaccttcccgatatatcacttacagttgacggaaacgactacgcagtgtgtgtaggctggagctgcttcg
MAM3–56	F	∆*pspABC* (upstream)	gaccagactcgctatttgtaac
MAM3–73	F	∆*pspBCDE* (flanking region 1)	agagatcaacaacggcgcattaagcgccgcggttagtatgagtattgagcaatagattatctatttttttactttcggcatatcaagacggtgtaggctggagctgcttcg
MAM3–31	R	∆*pspBCDE* (flanking region 2)	atgacaatggcggcgccgatacgcgccgtcgctcatcagctttaggtgtaaggagaacgcatgagcgcgctatttctggccatcccgttaatatgaatatcctcctta
MAM3–72	F	∆*pspBCDE* (upstream)	cgttattatcaactacccgg
MAM3–35	R	∆*pspBCDE* (downstream)	gtatcgatcaaatggaagcg
MAM3–46	F	pGEN-P*pspABCDE-luxCDABE*	gggggatccaattcaatcctcaca
MAM3–47	R	pGEN-P*pspABCDE-luxCDABE*	gggtacgtacaaagtgctcggcca
MAM3–52	F	pGEN-P*pspABCDE-S.bongori-ssrB-luxCDABE*	gggggatccaattcaatcctcacataatgttctggt
MAM3–57	F	pGEN-P*pspABCDE-scrambled-ssrB-luxCDABE*	gggggatccaattcaatcaattcaatcatagatctactactttcgaacgggcgat
MAM3–61	F	pGEN-MCS-*pspA*	ggggggaattcttattgattatcttgcttcattttggctttc
MAM3–62	R	pGEN-MCS-*pspA*	gtatgcggccgcgtaaaaccgggaaataagggcag

### RNA isolation of intracellular bacteria for sequencing

Wild-type SL1344 was enriched from infected bone marrow-derived macrophages (C57BL/6J) as previously described with modifications [18]. Macrophages were seeded 20 h prior to infection at ~10^7^ cells/100 cm^2^ tissue culture dish in RPMI containing 10% FBS (Gibco) with 100 ng/mL LPS from *S. enterica* serovar Minnesota R595 (Millipore). Overnight cultures of wild-type SL1344 were diluted to obtain a multiplicity of infection ~50:1 and added to each plate (wild-type invasion percentage of 5.15% ± 1.32). Plates were spun down at 500 x *g* for 2 min, then incubated for 20 min at 37°C with 5% CO_2_. Media was aspirated, plates were washed 3 times with PBS, and fresh RPMI with 10% FBS and 100 μg/mL gentamicin to kill extracellular bacteria was added. After a second incubation of 30 min at 37°C with 5% CO_2_, media was aspirated and replaced with fresh RPMI with 10% FBS and 10 μg/mL gentamicin. At this time (0 h) or 4 h, 8 h, and 12 h later (macrophage viability: 83.5% ± 3.92 after 12 h), media was aspirated, plates were washed twice with PBS, then infected macrophages were lysed on ice for 20 min in ice-cold 0.2% SDS, 1% acidic phenol, 19% ethanol in DEPC water. Lysates containing intracellular bacteria were collected and centrifuged at 4,000 rpm for 10 min, 4°C. After three washes in ice-cold wash buffer (1% acidic phenol, 19% ethanol in DEPC water) (4,000 rpm, 10 min, 4°C each wash), the supernatant was discarded, the bacterial pellet was resuspended in the remaining liquid, transferred to a clean RNAse-free microcentrifuge tube, then stored at -80°C. RNA was extracted using a MasterPure RNA purification kit (Epicentre) and treated with DNase I (Invitrogen). The quality of the purified RNA was verified with an Agilent RNA 6000 Pico BioAnalyzer.

### Sequencing, mapping of RNA-seq libraries, and differential gene expression analysis

All RNA-seq data are from three independent experiments per time-point. Prior to RNA-sequencing, ribosomal RNA was depleted using Ribo-Zero (Illumina), followed by the generation of barcoded cDNA for each sample. cDNA was sequenced on an Illumina NextSeq P2 platform with single-end reads. Raw reads were processed using FastQC [116] and trimmed with Trimmomatic [117] to remove Truseq adapter sequences. Sequencing data was mapped to the host reference genome GRCm39 (GCF_000001635.27) using BWA (mem algorithm) with default settings to remove non-bacterial sequences, then mapped to the reference genome of *S.* Typhimurium SL1344 (NC_016810) using BWA with default settings [118]. Uniquely-mapped reads were quantified using FeatureCounts [119] and the differential gene expression was determined using the R package DESeq2 [29]. The Likelihood ratio test (LTR) and reduced model parameters were used to identify genes that show changes in expression across all time points. Genes were deemed differentially regulated if they showed an FDR-adjusted *P* value < 0.01. For clustering analysis, regularized log transformation of the normalized counts of the differentially regulated genes were clustered using degPatterns from the R package DEGreport. The function was run using the default parameters and the produced clusters were visualized using the R package degPlotCluster [120]. Clusters of Orthologous Gene (COG) were assigned to significantly regulated genes using COGclassifier 1.0.5 Python package [121]. DESeq2 using the default parameters was used to conduct differential expression analysis of *S.* Typhimurium genes at early, middle, and late stages of infection relative to the onset stage. Genes were considered differentially regulated if they showed log_2_ fold change > 1 or <−1 at FDR-adjusted P value < 0.01. Circular visualization of the data was generated using the R package Circlize [122]. To generate the heatmap showing *psp* genes absolute expression, transcripts per million (TPM) values were calculated for all genes at each time point and values were then used to generate the heatmap using Pheatmap R package [123]. Raw sequencing reads have been deposited at Gene Expression Omnibus (GEO) database under series ID GSE294365.

### *In vitro* transcriptional reporter assays

Strains containing pGEN-*lux* promoter-reporter plasmid were grown in LB until the mid-log phase, then subcultured 1:50 into LPM media in black 96-well flat, clear-bottom plates (Corning). Plates were incubated at 37°C with shaking, and luminescence and OD_600_ were measured at 30-minute intervals for 16 h using an Agilent BioTek Cytation 5. Luminescence (RLU) was normalized to OD_600_.

### Growth curves

Overnight cultures of wild-type SL1344 or ∆*pspA* mutant were grown until mid-log phase in LB, then diluted 1:100 into LPM. OD_600_ was measured at 30 min intervals for 24 h using an Agilent BioTek Cytation 5.

### Intracellular replication assays

Differentiated bone marrow-derived macrophages (C57BL/6, C3H/HeN, or *Camp*^-/-^) were seeded 20 h prior to infection at 10^5^ cells/well in 96-well tissue culture plates in RPMI containing 10% FBS (Gibco) with 100 ng/mL LPS from *S. enterica* serovar Minnesota R595 (Millipore). Overnight cultures of wild-type SL1344, ∆*pspA* mutant, or ∆*pspBCDE* mutant were diluted to obtain a multiplicity of infection ~10:1 and added to each well (wild-type invasion percentage of 3.65% ± 1.16). Infected plates were spun for 2 min at 500 x *g* and incubated at 37°C with 5% CO_2_. Following 30 min of infection, bacteria-containing media was removed, macrophages were washed 3 times with PBS, and fresh RPMI with 10% FBS and 100 μg/mL gentamicin was added to each well to eliminate extracellular bacteria. Plates were incubated again for 30 min at 37°C with 5% CO_2_. Gentamicin-containing media was removed from macrophages and replaced with fresh RPMI with 10% FBS and 10 μg/mL gentamicin. Immediately after this media replacement step, adhered macrophages from 1/5 of the wells were washed twice with PBS and lysed in sterile water. Bacterial colony-forming units (CFUs) from each lysed well were enumerated by serially diluting in PBS and plating on LB plates supplemented with 34 μg/mL of chloramphenicol for ∆*pspA* and ∆*pspBCDE* mutants (CFU at 0 h). After 4 h, 8 h, 12 h, or 24 h of incubation at 37°C with 5% CO_2_ (macrophage viability: 80.0% ± 3.62 after 24 h), adhered macrophages from remaining wells were washed with PBS and lysed in sterile water for plating and CFU enumeration. Fold change in CFU (CFU at 4 h, 8 h, 12 h, or 24 h divided by 0 h) was calculated to represent replication throughout the experiment.

### Intramacrophage transcriptional reporter assays

Differentiated C57BL/6 bone marrow-derived macrophages were seeded 20 h prior to infection at 10^5^ cells/well in 96-well tissue culture-treated black 96-well flat, clear-bottom plates (Corning) in RPMI containing 10% FBS (Gibco) with 100 ng/mL LPS from *S. enterica* serovar Minnesota R595 (Millipore). Cells were infected with wild-type SL1344 harboring the empty pGEN*-luxCDABE* plasmid or the pGEN-P*pspABCDE-luxCDABE* reporter plasmid at a multiplicity of infection ~50:1 following the same procedure as used for the intracellular replication assays. Prior to collection and plating of intracellular bacteria at 0-, 4-, 8-, and 12-hour post-infection, luminescence was read at 450 nm using a PerkinElmer plate reader. Promoter activity was normalized to bacterial burdens by dividing RLU by CFU.

### Competitive infection

For bacterial preparation, wild-type SL1344 or ∆*pspABC* mutant strains were grown overnight in LB medium with appropriate antibiotic selection. For competitive infections, the inoculum consisted of a 1:1 ratio of wild-type strain resistant to streptomycin and a second competing mutant strain additionally resistant to chloramphenicol. Mice (C57BL/6 or C3H/HeN) were infected intraperitoneally (IP) with 2 x 10^5^ CFU bacteria in 0.1 M HEPES (pH 8.0) with 0.9% NaCl. After 45 hours mice were sacrificed, and total bacterial loads in the cecum, spleen, and liver were enumerated from organ homogenates serially diluted and plated on LB medium containing streptomycin. Colonies were replica-plated with chloramphenicol selection for the enumeration of mutant CFU. The competitive index was calculated using the following formula:


$$\frac{\left(\frac{mutant}{WT}\right)output}{\left(\frac{mutant}{WT}\right)input}$$

### Minimum inhibitory concentration assays

MIC determinations for PMB, colistin, CCCP, EDTA, polymyxin B nonapeptide, and CRAMP were performed using broth microdilution in 96-well flat, clear-bottom plates (Corning). Compounds were serially diluted two-fold starting at the following concentration: PMB and colistin; 100 μM, CCCP; 80μM, EDTA; 2 μM, PMBN, 50 μM, and CRAMP; 33 μM. Bacterial cultures grown overnight in LB were diluted ~1: 5 000 into LPM, then added to the compound-containing media. OD_600_ was read immediately after bacteria addition (OD_0h_) and after ~20 h of incubation at 37°C (OD_20h_) using a PerkinElmer plate reader. For the CRAMP MIC curve, percentage growth was calculated by subtracting OD_0h_ from OD_20h_, then normalizing values to a water control set to 100% growth.

### CRAMP, hydrogen peroxide, and spermine NONOate survival assays

Strains grown overnight in LB were subculture 1:50 into LPM and incubated at 37°C with shaking for 3 h. Bacteria were harvested and normalized to an OD_600_ of 0.5. Cells were washed and resuspended in LPM and diluted 1:10 (OD_600_ of 0.005). Each strain was incubated in LPM containing 10 μM of CRAMP, 50 μM of H_2_O_2_, or 50 μM of Spermine NONOate for 3 h at 37°C. The number of viable bacteria was determined by plating on LB agar supplemented with 34 μg/mL of chloramphenicol for ∆*pspA* and *∆phoP* strains, and percentage survival was calculated as the number of CFU/mL at 3 h relative to 0 h.

### DiSC_3_(5) assay

Wild-type SL1344 grown overnight in LB was subcultured 1:100 in LB or LPM media and incubated at 37°C for 4 h. Bacteria were harvested, washed twice with buffer (5 mM HEPES pH 7.0, 20 mM glucose), and resuspended to an OD_600_ of 0.1 in the same buffer. 100 mM KCl and 1 µM DiSC_3_(5) were added to the cell suspension and incubated in the dark at room temperature for 25 min. 148 µL of DiSC_3_(5)-loaded cells were then added to two-fold dilutions of valinomycin or CRAMP in 96-well black clear-bottom plates (Corning). Fluorescence was measured (Agilent BioTek Cytation 5) at an excitation wavelength of 620 nm and an emission wavelength of 685 nm at the start time and then every 70 s for 10 min and every 5 min for the next 20 min. For assays using the ∆*pspA* mutant, the same procedure was followed but DiSC_3_(5)-loaded cells (wild-type and mutant strains) were added to 1 µM or 100 µM of CRAMP.

### Data and statistical analysis

Data were analyzed using RStudio version 2024.04.2 + 764 with R version 4.4.1, and GraphPad Prism 10.1.1 software (GraphPad Inc., San Diego, CA). A *P* value of 0.05 or less was considered signiﬁcant. An explanation of the software used for RNA-sequencing analysis can be found in the corresponding experimental method description.

## Supporting information

S1 Table Summary of the data from the clustering analysis of the RNA-sequencing. DESeq2 performs a LTR test and reports P values that are adjusted for multiple testing using the procedure of Benjamini and Hochberg. Genes showing values showing adjusted *P* value < 0.01 were considered significant. Clustering was performed using degPatterns from the R package DEGreport. COG assignment data of all gene are reported as identified by COGclassifier. Cells with NA indicate that the gene could not be assigned to a particular COG family. Raw sequencing reads have been deposited at Gene Expression Omnibus (GEO) database under series ID GSE294365.
(XLSX)

S2 Table Summary of the data from the differential gene expression analysis of the RNA-sequencing. DESeq2 performs a Wald test and reports *P* values that are adjusted for multiple testing using the procedure of Benjamin and Hochberg. Genes showing values > 2 or <-2 for log_2_ fold change in expression at early, middle and late stages of infection relative to onset at adjusted *P* value < 0.01 were considered significant. Raw sequencing reads have been deposited at Gene Expression Omnibus (GEO) database under series ID GSE294365.
(XLSX)

S1 Fig The *pspABCDE* operon is expressed during host cell infection. Intramacrophage transcriptional reporter assay normalized to intracellular bacterial burdens. Bone marrow-derived macrophages were infected with wild-type (wt) *S.* Typhimurium harboring the *PpspABCDE-lux* transcriptional reporter construct or promotorless vector (control). Relative light units (RLU) were monitored over 12 hours at 0-, 4-, 8-, and 12-hours post-infection, and normalized to bacterial burdens. Bar plots depict mean of at three biological replicates (N = 3) and error bars indicate standard error of the mean. Groups were compared by two-way ANOVA, ****p < 0.0001 (Holm-Sidak’s multiple comparisons test); ns, not significantly different.
(TIF)

S2 Fig Identification of the SsrB putative binding site upstream of the *psp* operon. (A) Consensus motif logo of SsrB-binding site identified by Tomljenovic-Berube, *et. al.* (2013). (B) Aligned putative SsrB-binding site upstream of the *pspA* gene and known SsrB-regulated SPI-2 genes in *S.* Typhimurium. The left (7′) and right (7″) heptamers and the 4-bp spacer are displayed as a heat map to show bases of high conservation (dark blue) from degenerate regions (light blue/white). (C) Transcriptional reporter assay of the wild-type (wt) P*pspABCDE*-*lux* and the two mutated SsrB-binding site (putative *S. bongori* and scrambled SsrB-binding site) in wild-type *S.* Typhimurium grown in infection-mimicking media for 16h. Data are mean relative light units (RLU) normalized to the optical density of the culture from three independent experiments (N = 3).
(TIF)

S3 Fig Psp-deficient *S.* Typhimurium bacteria do not have a growth defect. (A) Growth of wild-type *S.* Typhimurium (wt) and *psp* mutants in rich media for 20 h. Data are from three biological replicates (N = 3), dots and error indicate mean and standard error of the mean. (B) As in (A), for wild-type and *psp* mutant strains in infection-mimicking media (LPM).
(TIF)

S4 Fig Inhibition of the Psp system attenuates *S.* Typhimurium for virulence in *NRAMP1*^+^ primary macrophages and mice model of infection. (A) Infection with wild-type (wt) or Δ*pspA* bacteria of bone marrow-derived macrophages harvested from C3H/HeN (*NRAMP1*^+^) mice. Intracellular survival was quantified as the bacterial burdens enumerated at 4, 8, 12, and 24 h following infection, normalized to the values of the initial number of internalized bacteria (T0). Bar plots depict the mean of three independent experiments (N = 3) and error bars indicate the standard of the mean. Groups were compared by two-way ANOVA, *p < 0.05, **p < 0.01 (Holm-Sidak’s multiple comparisons test). (B) Competitive infection of C3H/HeN mice. Mice were infected by intraperitoneal injection with equal numbers of wild-type (wt) *S.* Typhimurium and the Δ*pspABC* mutant strain, and the competitive index was calculated after 2 days of infection. Each data point is from an individual mouse, the horizontal lines indicate geometric means, and the broken line shows a competitive index of 1 (equal fitness). Data are from two independent experiments (N = 2). Groups were compared against a value of 1 using one-sample parametric T-test, *p < 0.05.
(TIF)

S5 Fig Complementation with *pspA* restores resistance to CRAMP. Growth in the presence of CRAMP in infection-mimicking media of *S.* Typhimurium ∆*pspA* mutant complemented with *pspA*, and strains carrying the empty pGEN-MCS vector control. Data are from at least two biological replicates (N ≥ 2), dots and error indicate mean and standard error of the mean.
(TIF)

S6 Fig Intracellular replication defect of *S.* Typhimurium ∆*pspA* bacteria is abrogated in *Camp*^-/-^ macrophages. (A) As in Figs 3A and 5C, intracellular survival of wild-type *S.* Typhimurium was measured as the ratio of the number of viable bacteria enumerated at 4, 8, 12, and 24 h compared to the initial number of internalized bacteria at T0 in C57BL/6J and *Camp*^*-/-*^ mice. (B) As in (A), but for *S.* Typhimurium ∆*pspA* mutant. Bar plots depict mean of at least four biological replicates (N ≥ 3) and error bars indicate standard error of the mean.
(TIF)
