# Supplementary figures and images for: The Salmonella phage shock protein system is required for defense against host antimicrobial peptides
Source: PLoS Pathog. 2025 Sep 9;21(9):e1013132. doi: 10.1371/journal.ppat.1013132 (PMC12440182; doi:10.1371/journal.ppat.1013132)

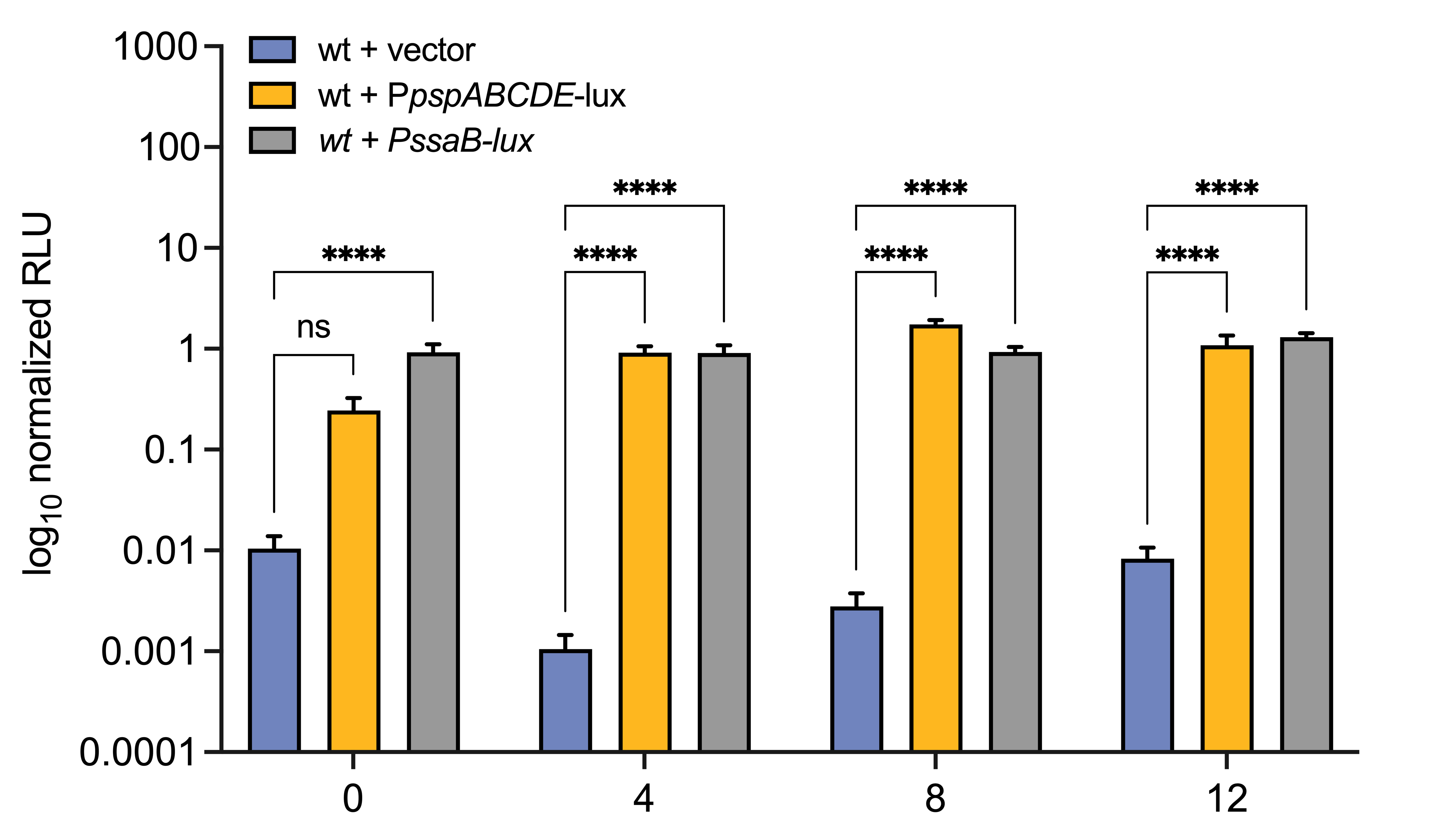

Supplement: S1 Fig — Intramacrophage transcriptional reporter assay normalized to intracellular bacterial burdens. Bone marrow-derived macrophages were infected with wild-type (wt) S. Typhimurium harboring the PpspABCDE-lux transcriptional reporter construct or promotorless vector (control). Relative light units (RLU) were monitored over 12 hours at 0-, 4-, 8-, and 12-hours post-infection, and normalized to bacterial burdens. Bar plots depict mean of at three biological replicates (N = 3) and error bars indicate standard error of the mean. Groups were compared by two-way ANOVA, ****p < 0.0001 (Holm-Sidak’s multiple comparisons test); ns, not significantly different. (TIF) [file ppat.1013132.s003.tif]

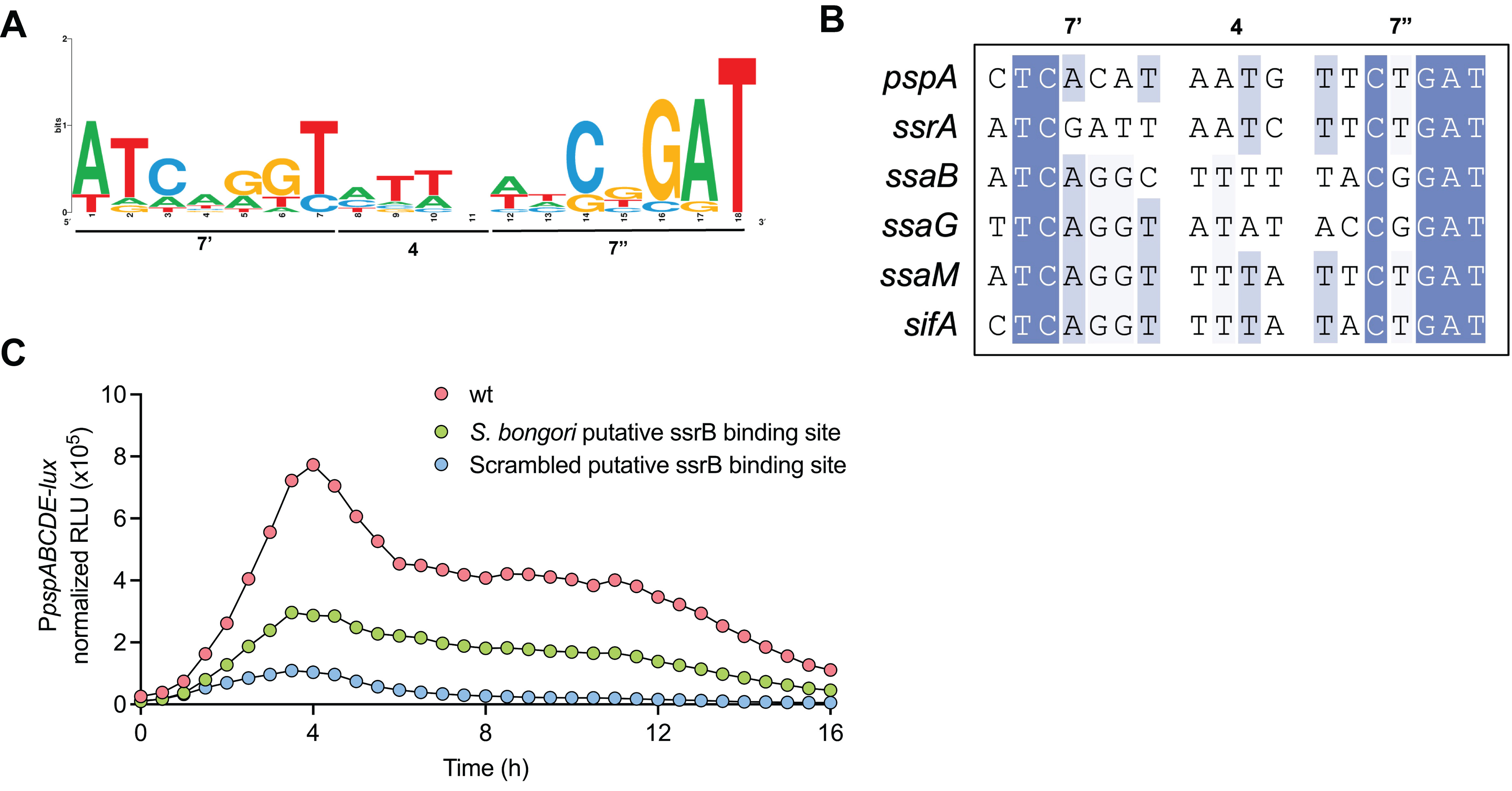

Supplement: S2 Fig — (A) Consensus motif logo of SsrB-binding site identified by Tomljenovic-Berube, et. al. (2013). (B) Aligned putative SsrB-binding site upstream of the pspA gene and known SsrB-regulated SPI-2 genes in S. Typhimurium. The left (7′) and right (7″) heptamers and the 4-bp spacer are displayed as a heat map to show bases of high conservation (dark blue) from degenerate regions (light blue/white). (C) Transcriptional reporter assay of the wild-type (wt) PpspABCDE-lux and the two mutated SsrB-binding site (putative S. bongori and scrambled SsrB-binding site) in wild-type S. Typhimurium grown in infection-mimicking media for 16h. Data are mean relative light units (RLU) normalized to the optical density of the culture from three independent experiments (N = 3). (TIF) [file ppat.1013132.s004.tif]

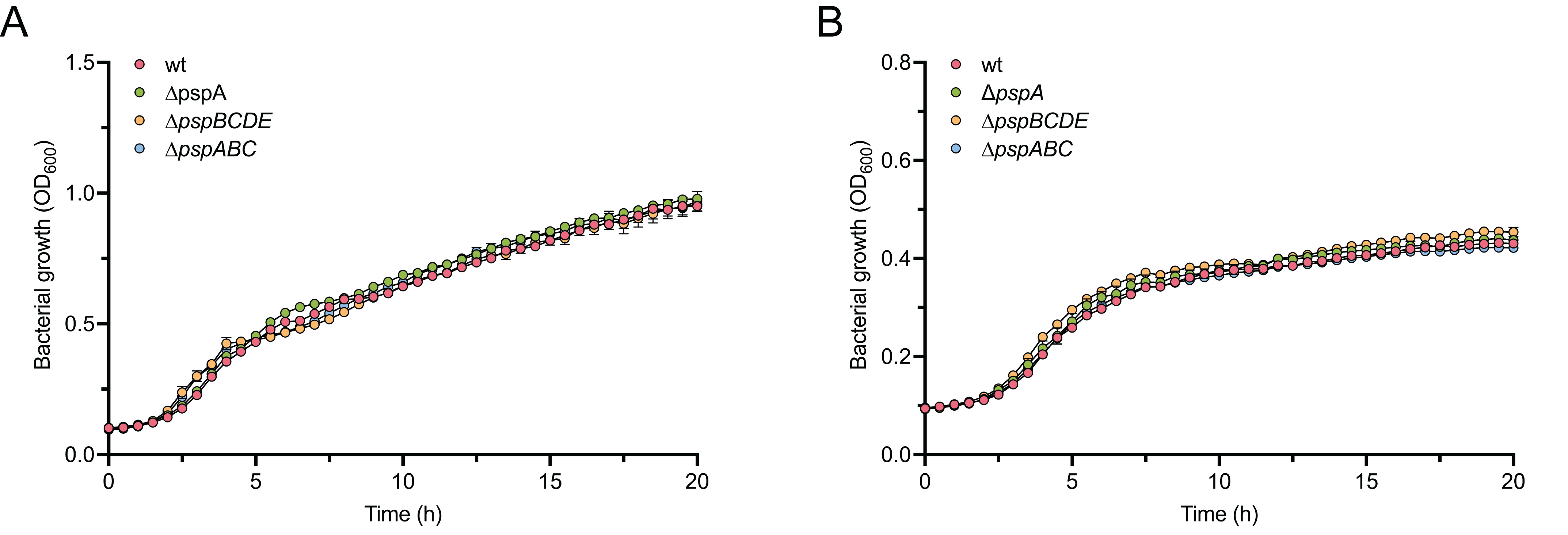

Supplement: S3 Fig — (A) Growth of wild-type S. Typhimurium (wt) and psp mutants in rich media for 20 h. Data are from three biological replicates (N = 3), dots and error indicate mean and standard error of the mean. (B) As in (A), for wild-type and psp mutant strains in infection-mimicking media (LPM). (TIF) [file ppat.1013132.s005.tif]

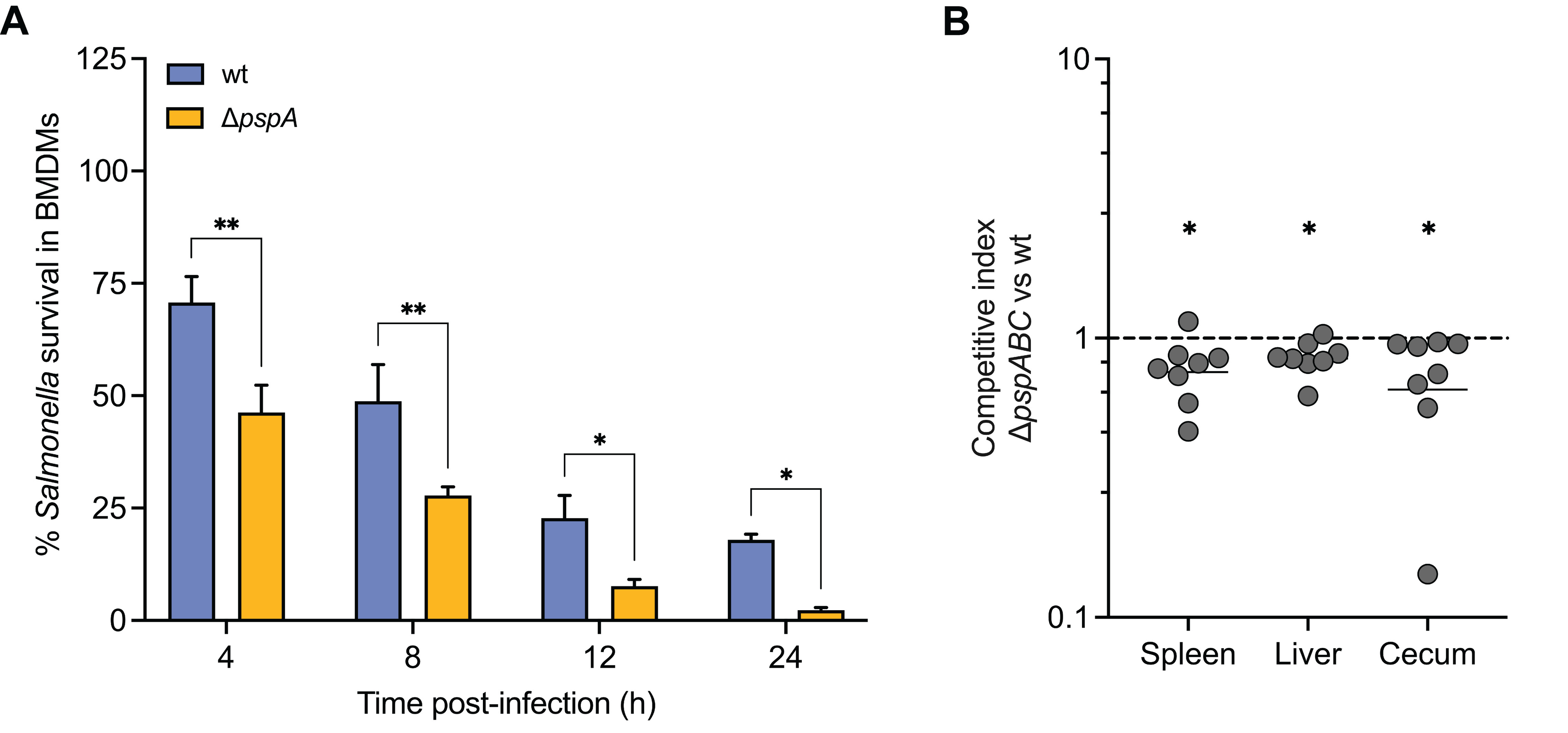

Supplement: S4 Fig — (A) Infection with wild-type (wt) or ΔpspA bacteria of bone marrow-derived macrophages harvested from C3H/HeN (NRAMP1+) mice. Intracellular survival was quantified as the bacterial burdens enumerated at 4, 8, 12, and 24 h following infection, normalized to the values of the initial number of internalized bacteria (T0). Bar plots depict the mean of three independent experiments (N = 3) and error bars indicate the standard of the mean. Groups were compared by two-way ANOVA, *p < 0.05, **p < 0.01 (Holm-Sidak’s multiple comparisons test). (B) Competitive infection of C3H/HeN mice. Mice were infected by intraperitoneal injection with equal numbers of wild-type (wt) S. Typhimurium and the ΔpspABC mutant strain, and the competitive index was calculated after 2 days of infection. Each data point is from an individual mouse, the horizontal lines indicate geometric means, and the broken line shows a competitive index of 1 (equal fitness). Data are from two independent experiments (N = 2). Groups were compared against a value of 1 using one-sample parametric T-test, *p < 0.05. (TIF) [file ppat.1013132.s006.tif]

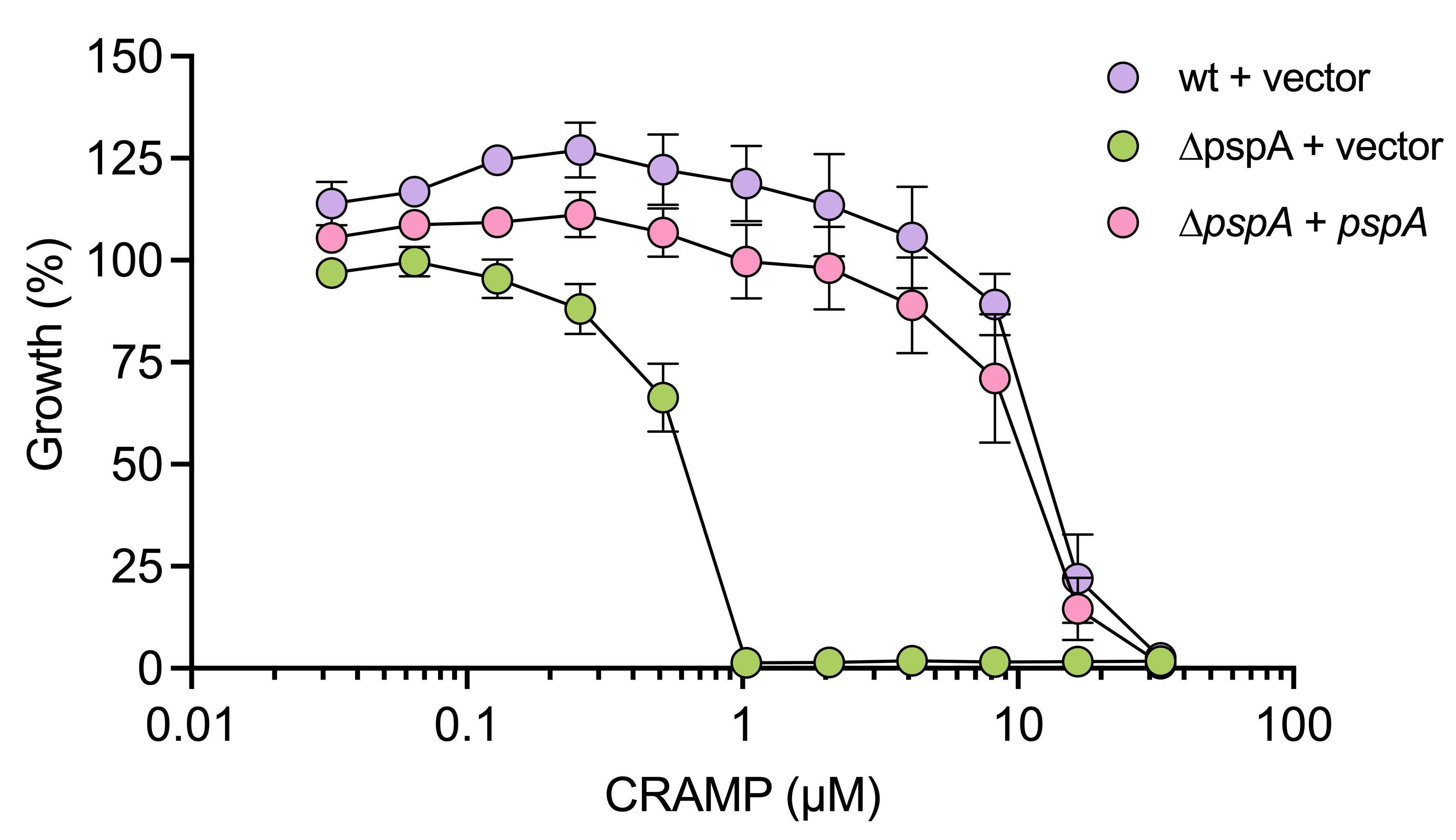

Supplement: S5 Fig — Growth in the presence of CRAMP in infection-mimicking media of S. Typhimurium ∆pspA mutant complemented with pspA, and strains carrying the empty pGEN-MCS vector control. Data are from at least two biological replicates (N ≥ 2), dots and error indicate mean and standard error of the mean. (TIF) [file ppat.1013132.s007.tif]

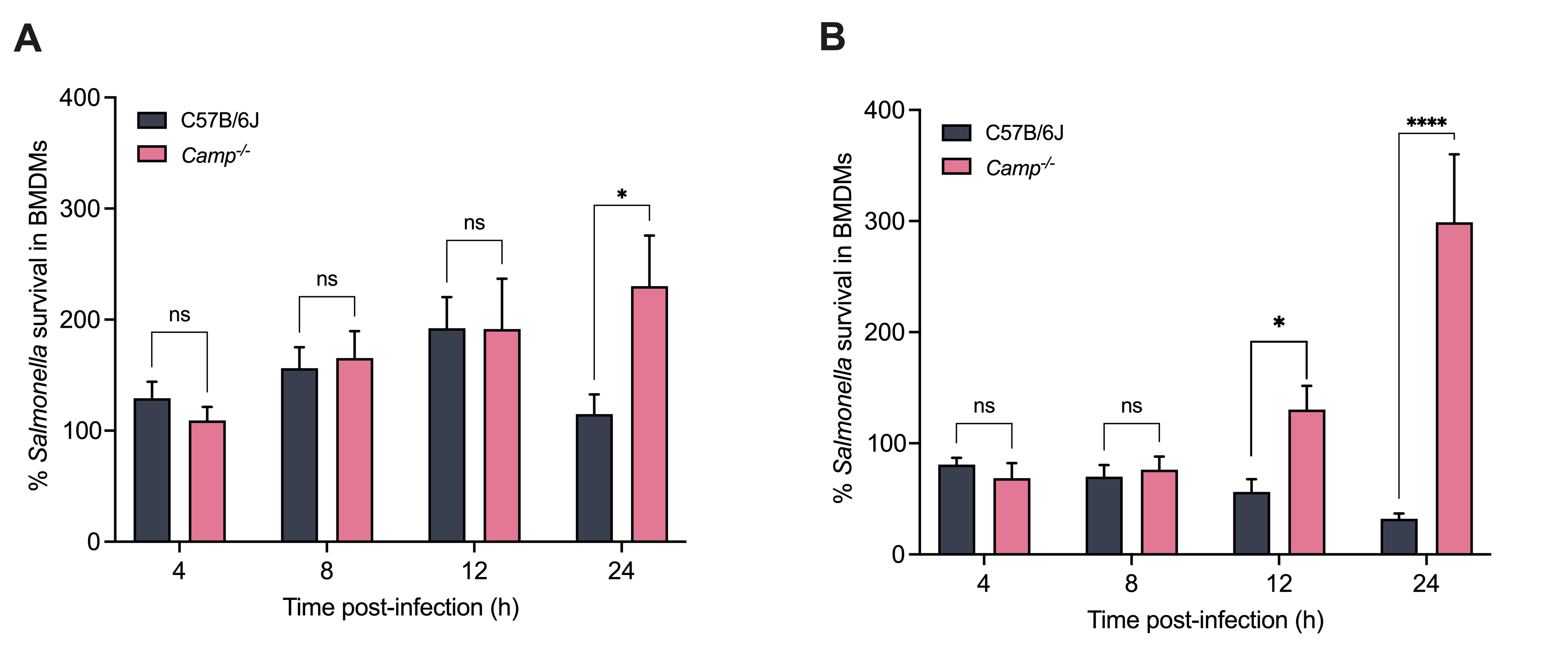

Supplement: S6 Fig — (A) As in Figs 3A and 5C, intracellular survival of wild-type S. Typhimurium was measured as the ratio of the number of viable bacteria enumerated at 4, 8, 12, and 24 h compared to the initial number of internalized bacteria at T0 in C57BL/6J and Camp-/- mice. (B) As in (A), but for S. Typhimurium ∆pspA mutant. Bar plots depict mean of at least four biological replicates (N ≥ 3) and error bars indicate standard error of the mean. (TIF) [file ppat.1013132.s008.tif]
